# Phosphatidylinositol phosphate kinase PIPKIγ and phosphatase INPP5E coordinate initiation of ciliogenesis

**DOI:** 10.1038/ncomms10777

**Published:** 2016-02-26

**Authors:** Qingwen Xu, Yuxia Zhang, Qing Wei, Yan Huang, Jinghua Hu, Kun Ling

**Affiliations:** 1 Department of Biochemistry and Molecular Biology, Mayo Clinic, Rochester, Minnesota 55905, USA; 2 Division of Nephrology and Hypertension, Mayo Clinic, Rochester, Minnesota 55905, USA

## Abstract

Defective primary cilia are causative to a wide spectrum of human genetic disorders, termed ciliopathies. Although the regulation of ciliogenesis is intensively studied, how it is initiated remains unclear. Here we show that type Iγ phosphatidylinositol 4-phosphate (PtdIns(4)P) 5-kinase (PIPKIγ) and inositol polyphosphate-5-phosphatase E (INPP5E), a Joubert syndrome protein, localize to the centrosome and coordinate the initiation of ciliogenesis. PIPKIγ counteracts INPP5E in regulating tau-tubulin kinase-2 (TTBK2) recruitment to the basal body, which promotes the removal of microtubule capping protein CP110 and the subsequent axoneme elongation. Interestingly, INPP5E and its product—PtdIns(4)P—accumulate at the centrosome/basal body in non-ciliated, but not ciliated, cells. PtdIns(4)P binding to TTBK2 and the distal appendage protein CEP164 compromises the TTBK2-CEP164 interaction and inhibits the recruitment of TTBK2. Our results reveal that PtdIns(4)P homoeostasis, coordinated by PIPKIγ and INPP5E at the centrosome/ciliary base, is vital for ciliogenesis by regulating the CEP164-dependent recruitment of TTBK2.

## Abstract (web summary)

The primary cilium is essential for embryonic development and tissue pattern formation. Here the authors show that PIPKIγ localizes to the basal body of the primary cilium and cooperates with the Joubert Syndrome associated protein INPP5E to regulate the initiation of ciliogenesis.

## Graphical abstract

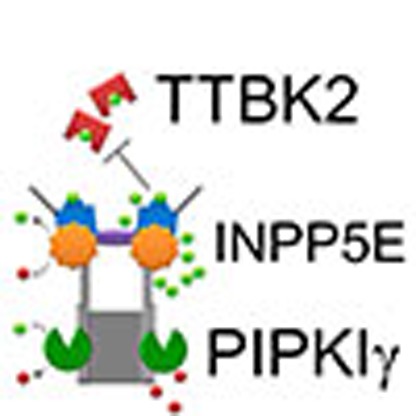

Primary cilia, the critical microtubule-based organelles that sense the environmental chemical and/or mechanical cues, regulate the homoeostasis of various organs/tissues in vertebrates^1^,^2^,^3^. While the overall biogenesis of the cilium is known, key aspects remain unclear. Early steps in ciliogenesis include the docking of the mother centriole (M-centriole)/basal body to the plasma membrane^4^,^5^,^6^, removal of the microtubule capping protein CP110 from the distal end of the M-centriole/basal body that allows the initiation of axoneme nucleation^7^, recruitment of intraflagellar transport (IFT) complexes^8^,^9^,^10^ and formation of a transition zone (TZ)^11^,^12^,^13^, followed by further extension of the microtubule axoneme and the ciliary membrane. Recruitment of TTBK2 to the M-centriole/basal body by the distal appendage/transition fibre (TF) protein CEP164 is essential for the removal of CP110 from the distal end of the M-centriole/basal body and the initiation of axoneme assembly^14^,^15^. However, the mechanism regulating TTBK2 recruitment remains elusive.

Phosphoinositides (PIs) are generated by phosphorylation of phosphatidylinositol (PtdIns) at the 3, 4 and/or 5 positions of the inositol ring. By the spatially localized recruitment of effector proteins^16^,^17^, PI species regulate various important membrane/protein trafficking or cytoskeleton-related cellular processes throughout the cell^18^,^19^. Protein recruitment often occurs through a specific PI-binding domain on the protein. For instance, pleckstrin homology (PH)^20^,^21^, Phox homology, Fab1, YOTB, Vac 1, EEA1 (FYVE), epsin N-terminal homologue domains and so on, as well as less-conserved basic motifs, bind to PIs with varying affinities and specificities^16^,^22^. PI binding usually mediate the targeting of the protein to a specific membrane compartment and/or induce a conformational change that regulates the interaction between the protein and its binding partner^16^. Lately, a few studies implicated PI signalling in the context of cilia and ciliopathies. For example, three PI 5-phosphatases localize to cilia and are correlated with ciliopathies (that is, INPP5E in Joubert syndrome and nephronophthisis^23^,^24^, and OCRL and INPP5B in Lowe syndrome^25^,^26^). PtdIns(4,5)P_2_, a substrate of INPP5E, is required for flagella outgrowth during *Drosophila* spermatogenesis^27^ and reduced in rodent polycystic kidney disease models^28^,^29^. Recently, two groups independently reported that INPP5E localizes in primary cilia and maintains a PtdIns(4)P-high, PtdIns(4,5)P_2_-low environment, which ensures the processing of hedgehog signalling by inhibiting the ciliary entry of TULP3 and Gpr161 (refs 30, 31). These studies clearly support the importance of PIs in ciliary signalling; however, whether and how these phospholipids participate in ciliogenesis is unclear.

In current study, we show that PIPKIγ, a centrosomal PtdIns(4)P 5-kinase^32^, presents at the basal body in ciliated cells. INPP5E also resides at the M-centriole in serum-fed, non-ciliated cells; however, translocates to cilium proper in ciliated cells. We find that PIPKIγ is required for, but INPP5E inhibits, ciliogenesis. Consistently, these two PI enzymes regulate the recruitment of TTBK2 to the M-centriole in an opposite manner. Further investigation demonstrates a centrosomal pool of PtdIns(4)P, a product of INPP5E and the substrate of PIPKIγ, prevents the recruitment of TTBK2 to the M-centriole by binding to CEP164 and TTBK2, and inhibiting the TTBK2-CEP164 interaction. Overall, these discoveries reveal a novel mechanism that PIPKIγ and INPP5E coordinate the initiation of ciliogenesis by spatiotemporally regulating a centrosomal PtdIns(4)P pool to control the TTBK2 recruitment and CP110 removal.

## Results

### PIPKIγ at the basal body is necessary for ciliogenesis

**Figure 1:**
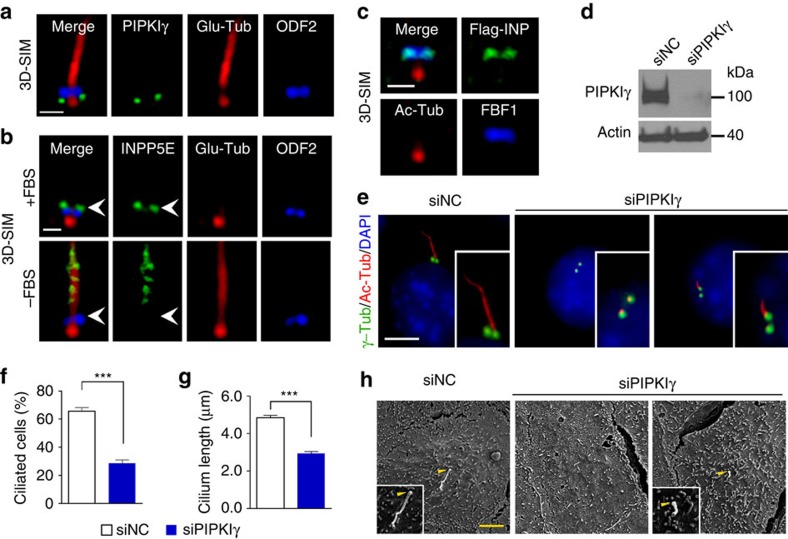
PIPKIγ is required for ciliogenesis. (**a**–**c**) RPE-1 cells were treated as described below, subjected to indirect immunofluorescence (IF) labelling with indicated antibodies, and than analysed with three-dimensional structured illumination microscopy (3D-SIM). (**a**) Serum-starved RPE-1 cells; (**b**) RPE-1 cells before (+FBS) and after (−FBS) 24 h serum starvation; (**c**) RPE-1 cells expressing Flag-tagged INPP5E (Flg-INP).Ac-Tub, acetylated α-tubulin; Glu-Tub, polyglutamylated tubulin. (**d**) IMCD3 cells were treated with negative control (siNC) or PIPKIγ-specific (siPIPKIγ) siRNAs for 48 h. (**e**–**g**) IMCD3 cells described in **d** were serum starved for 24 h and subjected to IF. Nuclei were visualized by DAPI staining (blue). Insets show magnified images of primary cilia. γ-Tub, γ-tubulin. Percentage of ciliated cells (**f**) and cilium length (**g**) in each group (*n*>200) were quantified from three independent experiments and plotted. Error bars represent s.d. in **f** and s.e.m. in **g**. ***P<0.001. (**h**) Cells described in **d** were analysed by scanning electron microscopy. Arrowheads indicate the ciliary base. Scale bar, 0.5 μm in **a**–**c**; Scale bar, 5 μm in **e** and **h**.

Our previous work describing the function of PIPKIγ at the centrosome^32^ suggests that PIPKIγ might also participate in the context of cilia when the M-centriole transforms into the basal body. To test this, we first investigated the subcellular localization of PIPKIγ in ciliated cells using a validated anti-PIPKIγ antibody^32^. As shown in Fig. 1a, PIPKIγ was visualized at the ciliary base in human retinal pigment epithelial (RPE-1) cells, but not in cells treated with PIPKIγ-specific siRNAs (Supplementary Fig. 1a,b). We further confirmed this basal-body localization of PIPKIγ in other types of ciliated cells, including mouse inner medullary collecting duct (IMCD3) cells, NIH3T3 cells and human renal cortical tubular epithelial (RCTE) cells (Supplementary Fig. 1c). Images obtained by super-resolution three-dimensional structured illumination microscopy revealed that PIPKIγ localizes below the distal end marker ODF2 in RPE-1 cells (Fig. 1a) and forms a ring-like structure with similar diameter as the centrosome protein PLK4 (Supplementary Fig. 1f). Meanwhile, PIPKIα and PIPKIβ antibodies yielded no cilium-related signal (Supplementary Fig. 1d,e), indicating that PIPKIγ is the sole type I PIP kinase at the basal body.

Recent studies have implicated PI 5-phosphatase INPP5E in the context of cilia^23^,^30^,^31^,^33^; thus, we examined the spatial relationship between INPP5E and PIPKIγ. Distinct from the proximal localization pattern for PIPKIγ, INPP5E resides slightly above ODF2 (Fig. 1b, upper panels) and colocalizes with the distal appendage protein FBF1 (Fig. 1c) on the M-centriole in non-ciliated cells. In ciliated cells, INPP5E is no longer detected at the basal body (Fig. 1b, lower panels), and presents only in the ciliary lumen as reported^23^,^33^. Considering PIPKIγ and INPP5E are counteracting enzymes, these results suggest that the level of corresponding PIs (PtdIns(4)P and/or PtdIns(4,5)P_2_) around the M-centriole/basal body ought to be changed before and after ciliogenesis.

We next sought to determine the physiological importance of PIPKIγ at the ciliary base. Using the specific siRNAs^32^, we found that knockdown of PIPKIγ, but not PIPKIα or PIPKIβ, significantly inhibited ciliogenesis in various types of mammalian cells (Fig. 1d–g; Supplementary Fig. 1g–i). In the small portion of PIPKIγ-depleted cells that still formed cilia, the average cilium length was about half of that in control cells (Fig. 1e,g). These phenotypes were confirmed by scanning electron microscopy (Fig. 1h). Interestingly, *ppk-1* (the solo *PIPKI* in *Caenorhabditis elegans*)-knockout nematodes also exhibit defective ciliogenesis (Supplementary Fig. 1j,k), suggesting the function of PIPKIγ in ciliogenesis likely evolves early across ciliated species.

### Loss of PIPKIγ affects TZ but not TF assembly

**Figure 2:**
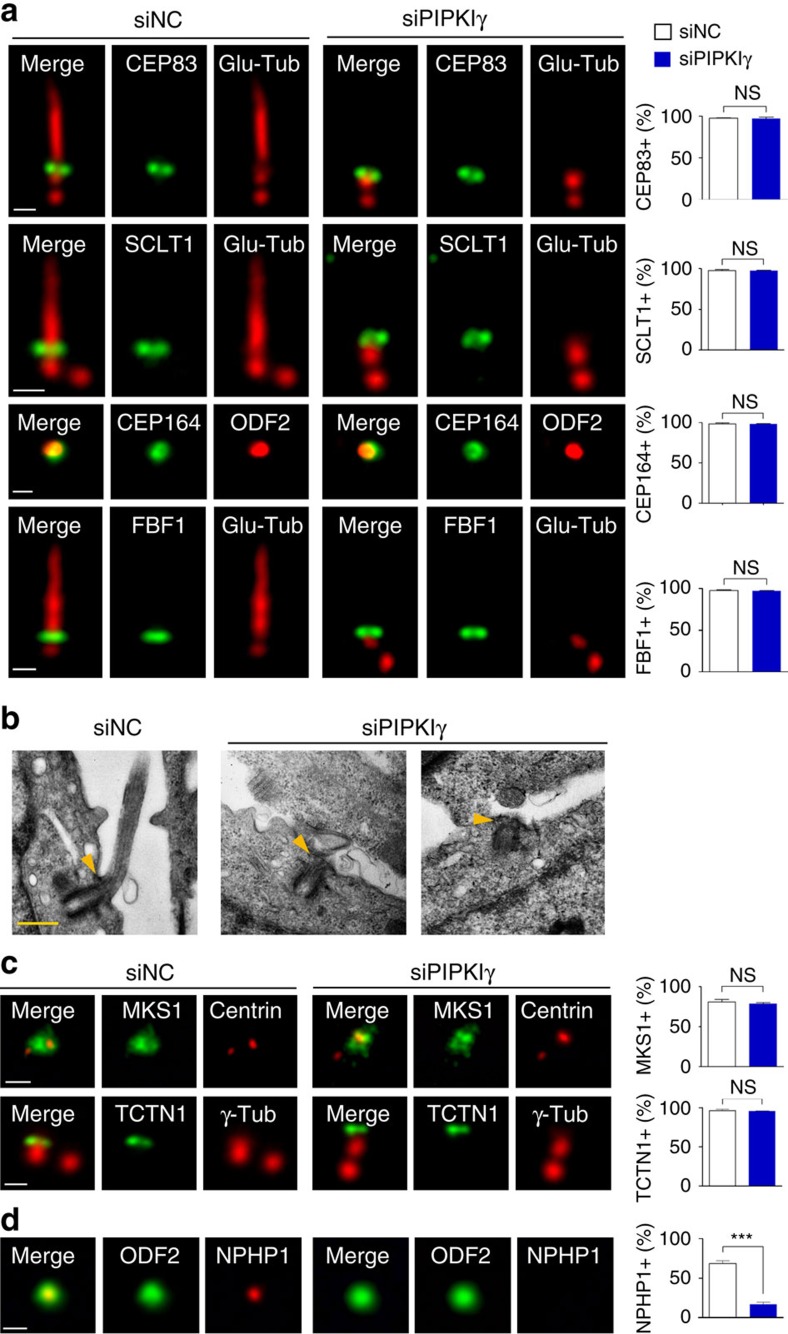
PIPKIγ-depleted cells exhibit normal basal body docking to the plasma membrane. (**a**,**c**,**d**) RCTE cells were treated with negative control (siNC) or PIPKIγ-specific (siPIPKIγ) siRNAs for 48 h and serum starved for 24 h, then subjected to indirect IF microscopy analyses with indicated antibodies. Glu-Tub, polyglutamylated tubulin; γ-Tub, γ-tubulin. Scale bars, 0.5 μm. (**a**) The localization of transition fibre proteins is not affected by PIPKIγ depletion. (**b**) The basal body docking to the plasma membrane is not changed by PIPKIγ depletion. Control (siNC) or PIPKIγ-depleted (siPIPKIγ) IMCD3 cells grown on coverslips were serum starved for 48 h and then fixed, scraped off the coverslips, spun into pellets and processed for transmission electron microscopy. Arrowheads point to distal ends of the basal body. (**c**) Localization of the components of transition zone MKS module is not affected by PIPKIγ depletion. (**d**) NPHP1 lost its association with the M-centriole/basal body in PIPKIγ-depleted cells. Percentage of cells (*n*>60) exhibiting centrosome/basal body localization of tested transition fibre (**a**) or transition zone (**c**,**d**) proteins in control or PIPKIγ-depleted cells was quantified from three independent experiments and plotted. Error bars represent s.d. NS, no statistically significant difference. ***P<0.001.

To understand how PIPKIγ regulates ciliogenesis, we carefully examined PIPKIγ-depleted cells. TFs, which are transformed from the distal appendages of the M-centriole, are important for tethering the basal body to the plasma membrane^4^,^5^ and docking the IFT particles to the basal body^34^. In PIPKIγ-depleted RCTE (Fig. 2a) and RPE-1 (Supplementary Fig. 2) cells, TF components (CEP83, SCLT1, CEP164 and FBF1) maintain their regular localizations at the ciliary base. Consistently, PIPKIγ-depleted cells displayed normal basal body docking to the plasma membrane (Fig. 2b), which also suggests that TFs are formed properly when PIPKIγ is absent. We then inspected the localization of TZ proteins. Interestingly, although the localization of MKS components MKS1 and TCTN1 was not affected by depletion of PIPKIγ, no NPHP1 was detected on the basal body (Fig. 2c,d; Supplementary Fig. 2), indicating that NPHP1 recruitment is interrupted. These data suggest that PIPKIγ acts after basal body docking and before TZ formation. Capping protein CP110 suppresses microtubule nucleation; therefore it must be removed from the M-centriole to allow TZ assembly and axoneme elongation when ciliogenesis is induced^7^. Thus, we next examined whether PIPKIγ is required for CP110 removal.

### PIPKIγ regulates for TTBK2 recruitment and CP110 removal

**Figure 3:**
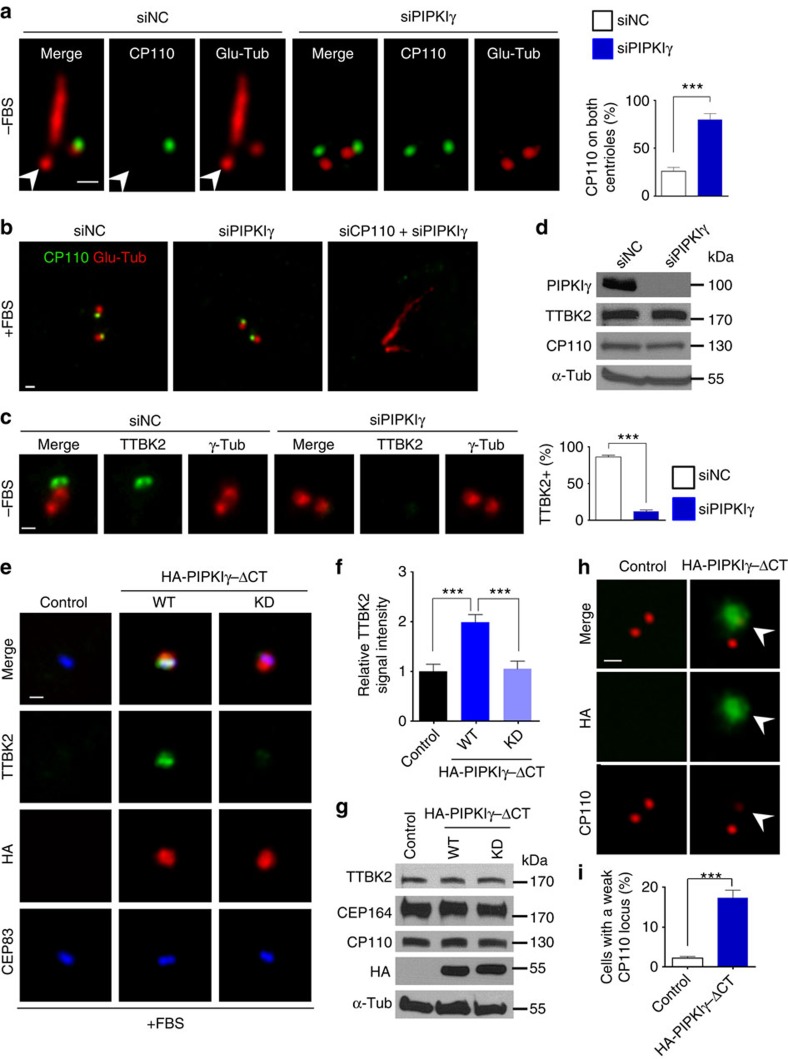
PIPKIγ is required for the removal of CP110 from and the recruitment of TTBK2 to the M-centriole/basal body. (**a**,**c**) RCTE cells were treated with control (siNC) or PIPKIγ-specific (siPIPKIγ) siRNA for 48 h, serum starved (−FBS) for additional 24 h and stained with indicated antibodies for IF microscopy. Percentage of cells (*n*>200) with CP110 on both centrioles (**a**) or with TTBK2 at the M-centriole/basal body (**c**) was quantified from three independent experiments. Error bars represent s.d. (**b**) Serum-fed (+FBS) RCTE cells were treated with siNC, siPIPKIγ or siPIPKIγ plus CP110-specific (siCP110) siRNA for 72 h, and then subjected to IF microscopy to visualize polyglutamylated tubulin and CP110. (**d**) CP110 and TTBK2 protein levels are not changed in PIPKIγ-depleted cells. Cell lysates from control (siNC) or PIPKIγ-depleted (siPIPKIγ) cells were immunoblotted using indicated antibodies. (**e**) Serum-fed RCTE cells stably expressing GFP-TTBK2 were transfected with empty vector (Control), wild-type (WT) or kinase-dead (KD) HA-PIPKIγΔCT for 8 h. Then cells were subjected to IF microscopy to visualize TTBK2, HA tag and CEP83. (**f**) The fluorescence intensity of centrosomal TTBK2 in >20 control, HA-PIPKIγΔCT-WT or HA-PIPKIγΔCT-KD expressing cells was quantified from three independent experiments and plotted. Error bars represent s.e.m. (**g**) Cell lysates from cells described in **e** were immunoblotted using indicated antibodies. α-Tub, α-tubulin. (**h**) CP110 was visualized by IF microscopy in serum-fed control or HA-PIPKIγΔCT-WT expressing cells. (**i**) Percentage of cells (*n*>40) showing diminished CP110 signal at M-centrioles was quantified in each group and results from three independent experiments were plotted. Error bars represent s.d. Arrowheads indicate M-centrioles/basal bodies. ***P<0.001. Scale bars, 0.5 μm.

Remarkably, in PIPKIγ-depleted cells, CP110 is abnormally preserved on both centrioles on serum starvation (Fig. 3a; Supplementary Fig. 3a). Depletion of CP110 restores the axoneme elongation in PIPKIγ-deficient cells (Fig. 3b), suggesting that PIPKIγ functions upstream of CP110 during ciliogenesis. It is known that the removal of CP110 depends on the recruitment of TTBK2 to the M-centriole/basal body on serum starvation or growth inhibition^14^. In agreement with the sustained CP110, we found no TTBK2 at the M-centriole/basal body after serum starvation in PIPKIγ-depleted cells (Fig. 3c; Supplementary Fig. 3b). In the context that the global protein levels of TTBK2 and CP110 were not changed by PIPKIγ depletion (Fig. 3d), we propose that PIPKIγ is required for the serum starvation-induced translocation of TTBK2 to the M-centriole/basal body, which subsequently promotes the removal of CP110, and then TZ assembly and axoneme elongation.

Since PIPKIγ is a lipid kinase, we then asked whether its enzyme activity is required for the recruitment of TTBK2 to the M-centriole. As reported, the C-terminus-truncated PIPKIγ (PIPKIγΔCT) exhibits specific targeting to centrioles with stronger association with the M-centriole^32^, unlike the full-length PIPKIγ that also localizes to other subcellular locales such as the plasma membrane, cell adhesions and vesicle compartments^35^,^36^. Therefore, we utilized PIPKIγΔCT to eliminate phenotypes unrelated to primary cilia. Notably, overexpression of the active PIPKIγΔCT, but not the kinase-dead mutant (D253/316A), can promote the recruitment of TTBK2 to the M-centriole even without serum starvation (Fig. 3e–g), indicating that excessive PIPKIγ kinase activity stimulates the recruitment of TTBK2 to the M-centriole independent of serum starvation. These effects were reproduced in cells overexpressing the full-length wild-type or kinase-dead PIPKIγ (Supplementary Fig. 3c). Accompanied with the increased TTBK2 at the centrosome, CP110 signal at the M-centriole was substantially decreased but not entirely vanished in cells ectopically expressing PIPKIγΔCT (Fig. 3h,i), suggesting that the TTBK2 recruited to the M-centriole by overexpressed PIPKIγΔCT is functional but not sufficient to completely remove CP110. Indeed, ectopically expressed PIPKIγΔCT did not induce serum starvation-independent ciliogenesis (Supplementary Fig. 3d).

### INPP5E inhibits TTBK2 recruitment and CP110 removal

**Figure 4:**
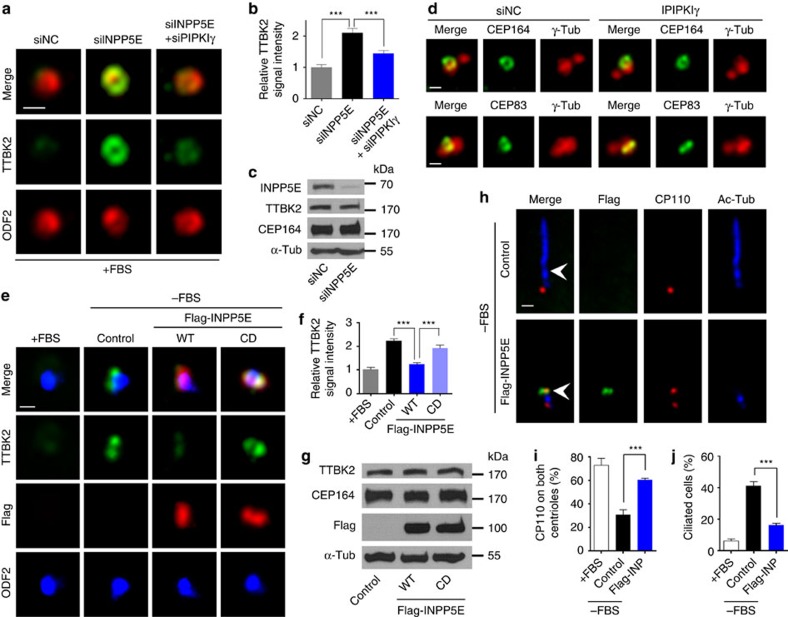
INPP5E inhibits the association of TTBK2 with the M-centriole/basal body. (**a**,**b**) TTBK2 was visualized by IF microscopy in serum-fed RCTE cells 72 h post transfection of the negative control (siNC), INPP5E-specific (siINPP5E) and/or PIPKIγ-specific (siPIPKIγ) siRNAs. ODF2 was used as a centrosome marker. (**b**) The centrosomal TTBK2 intensity in cells (*n*>30) of each group was quantified and plotted from three independent experiments. (**c**,**d**) RCTE cells treated with siNC or siINPP5E were collected for immunoblotting (**c**) or IF microscopy (**d**) with indicated antibodies. α-Tub, α-tubulin. (**e**,**f**) RCTE cells were transfected with empty vector (Control), wild type (WT) or catalytic defective (CD) Flag-INPP5E for 8 h, and serum starved for additional 8 h before stained with indicated antibodies. In each group, the intensity of centrosomal TTBK2 signal in >30 cells was quantified from at least three independent experiments and plotted. Error bars represent s.e.m. (**g**) Cells transfected with empty vector (Control), WT or CD Flag-INPP5E were collected for immunoblotting with indicated antibodies. (**h**–**j**) RCTE cells transfected with empty vector (Control) or wild-type Flag-INPP5E for 24 h were serum-starved for additional 36 h, and then stained with appropriate antibodies. Percentage of cells with CP110 on both centrioles (**i**; *n*>100) or with primary cilium (**j**; *n*>200) was quantified in each group. Results from at least three independent experiments were plotted. Error bars represent s.d. ***P<0.001. Scale bars, 0.5 μm.

Our results suggest that the kinase activity of PIPKIγ at the M-centriole/basal body is indispensable for the early stage of ciliogenesis. Therefore, we expect that INPP5E, which localizes at the centrosome in serum-fed cells and potentially counteracts PIPKIγ on site, would neutralize PIPKIγ function in TTBK2 recruitment. Indeed, knockdown of INPP5E led to a substantial increase of TTBK2 signal on the M-centriole in serum-fed cells (Fig. 4a–c) without affecting the localization patterns of the TF proteins CEP164 and CEP83 (Fig. 4d). This TTBK2 accumulation triggered by INPP5E depletion was significantly suppressed by co-depleting PIPKIγ (Fig. 4a,b). On the other hand, overexpression of the wild-type INPP5E almost completely inhibited TTBK2 recruitment in response to serum starvation, whereas the catalytic-defective INPP5E (D512/515W) related to Joubert syndrome^33^ had no such effect (Fig. 4e–g), indicating that the activity of INPP5E is required to inhibit TTBK2 recruitment. As a consequence of impaired TTBK2 recruitment to the M-centriole, serum-starvation-induced CP110 removal and ciliogenesis were both inhibited in INPP5E overexpressing RCTE cells (Fig. 4h–j) as well as IMCD3 cells (Supplementary Fig. 4a,b). In summary, the alterations of TTBK2 recruitment and CP110 removal caused by decreasing or increasing INPP5E activity (Fig. 4a,e) recapitulated those caused by increasing or decreasing PIPKIγ activity (Fig. 3c,e), respectively. This leads to a conclusion that PIPKIγ and INPP5E oppositely regulate the recruitment of TTBK2 to the M-centriole during the initiative step of ciliogenesis.

### A centrosomal PtdIns(4)P pool inhibits TTBK2 recruitment

**Figure 5:**
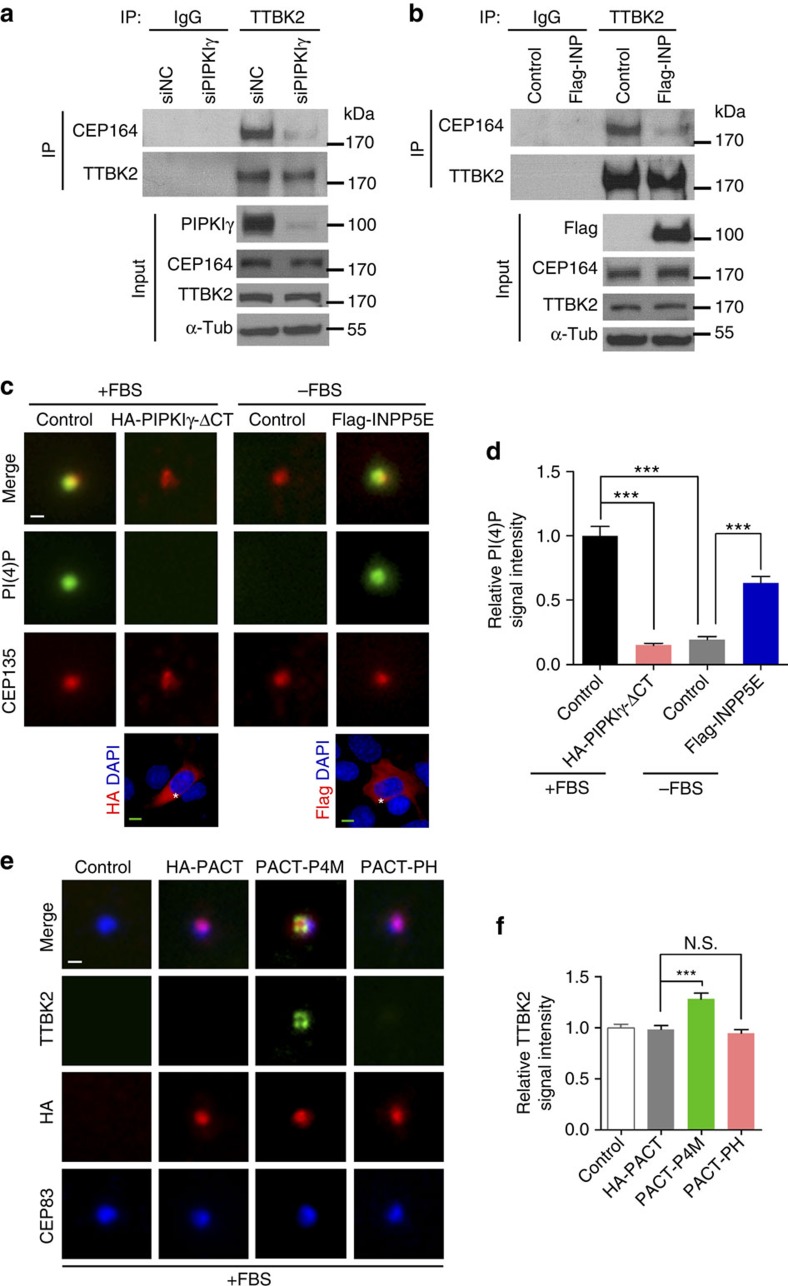
PIPKIγ and INPP5E coordinate the association between TTBK2 and CEP164 by regulating a centrosomal PtdIns(4)P pool. (**a**,**b**) HEK293T cells transfected with indicated siRNAs (**a**) or plasmids (**b**) were subjected to immunoprecipitation (IP) with anti-TTBK2 antibody or normal rabbit IgG (IgG). Flag-INP, Flag-INPP5E. The resulting precipitates and cell lysates (Input) were immunoblotted with indicated antibodies. α-Tub, α-tubulin. (**c**) IMCD3 cells were transfected with empty vector (Control), HA-PIPKIγΔCT or Flag-INPP5E. Without (+FBS) or with 6- h serum starvation (−FBS, most cells were not yet ciliated under this condition), each group of cells was subjected to IF microscopy to visualize PtdIns(4)P (PI(4)P, green), CEP135 (red), HA-tag (far-red) and nuclei (blue, DAPI staining). Stars indicate transfected cells. (**d**) In each group in **c**, the intensity of centrosomal PtdIns(4)P signal in >20 cells was quantified and plotted from at least three independent experiments. (**e**,**f**) RCTE cells stably expressing GFP-TTBK2 were transfected with HA-tagged PACT, PACT-P4M^SidM^ (PACT-P4M) or PACT-PH^PLCδ^ (PACT-PH) for 24 h. Cells were then processed for IF microscopy with antibodies against TTBK2 (green), HA-tag (red) and CEP83 (far-red, pseudo-colored blue). (**f**) The fluorescence intensity of centrosomal TTBK2 was quantified in >20 cells of each group. Results from three independent experiments were plotted. Error bars represent s.e.m. ***P<0.001. Scale bars, 5 μm (green); Scale bars, 0.5 μm (white).

It was reported that binding to CEP164 is required for TTBK2 recruitment to the M-centriole/basal body^37^. Because the protein level of either TTBK2 or CEP164 is not affected by manipulating the activity of PIPKIγ (Fig. 3d,g) or INPP5E (Fig. 4c,g), we reason that the association between TTBK2 and CEP164 might be disturbed. As expected, both PIPKIγ depletion (Fig. 5a) and INPP5E overexpression (Fig. 5b) led to substantially reduced association between TTBK2 and CEP164. These results, together with our data that the activities of PIPKIγ (Fig. 3e–g) and INPP5E (Fig. 4e–g) are required for regulating TTBK2 recruitment, suggest that PtdIns(4)P (substrate of PIPKIγ and product of INPP5E) and/or PtdIns(4,5)P_2_ (product of PIPKIγ and substrate of INPP5E) may regulate the interaction between TTBK2 and CEP164. To test this, we first determined whether PtdIns(4)P or PtdIns(4,5)P_2_ presents at the centrosome. As shown in Fig. 5c validated PtdIns(4)P antibody^30^,^31^ (Supplementary Fig. 5a) nicely decorates the centrosome in serum-fed, non-ciliated cells; however, we did not observe specific centrosome accumulation of PtdIns(4,5)P_2_, PtdIns(3)P or PtdIns(5)P (Supplementary Fig. 5b–d). The centrosomal PtdIns(4)P signal diminished when PIPKIγ was overexpressed or serum starvation was applied (Fig. 5c,d), both conditions induce the TTBK2 recruitment to the M-centriole. Furthermore, ectopic expression of the wild-type INPP5E restored PtdIns(4)P at the centrosome in serum-starved cells (Fig. 5c,d). These results, confirming the authenticity of this centrosomal PtdIns(4)P pool, indicate a negative correlation between the accumulation of PtdIns(4)P and the recruitment of TTBK2 at the centrosome. To further investigate whether this centrosomal PtdIns(4)P pool is physiologically relevant to the TTBK2 recruitment, we fused the pericentrin-AKAP450 centrosomal targeting (PACT) domain^38^ to the PtdIns(4)P-binding P4M domain of SidM^39^ or PtdIns(4,5)P_2_-binding PH domain of PLCδ^40^. Then, we overexpressed these proteins in cells to achieve the spatial sequestration of PtdIns(4)P or PtdIns(4,5)P_2_ at the centrosome, respectively. Remarkably, only the PACT-P4M^SidM^, but not the PACT alone or PACT-PH^PLCδ^, resulted in an accumulation of TTBK2 at the centrosome in serum-fed cells (Fig. 5e,f), indicating that the depletion of centrosomal PtdIns(4)P favours TTBK2 recruitment *in vivo*. Considering the loss of PIPKIγ and increase of INPP5E, both diminish the association between TTBK2 and CEP164 (Fig. 5a,b), we propose that PtdIns(4)P inhibits the TTBK2–CEP164 interaction.

### PtdIns(4)P inhibits the CEP164–TTBK2 interaction

**Figure 6:**
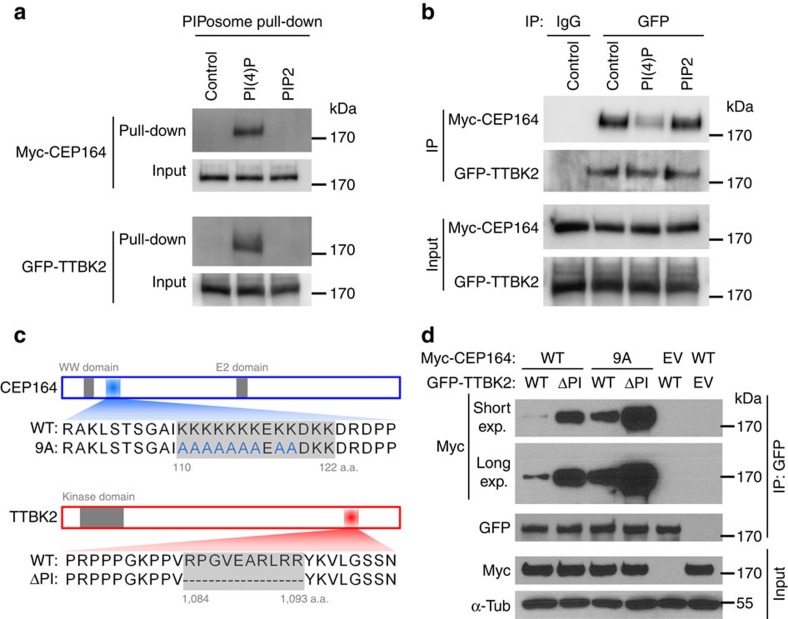
PtdIns(4)P binds to CEP164 and TTBK2 and inhibits their interaction *in vitro*. (**a**) Cell lysates prepared from HEK293T cells overexpressing Myc-CEP164 or GFP-TTBK2 were subjected to PolyPIPosome (PIPosome) pull-down assay with control PolyPIPosomes or PolyPIPosomes containing PtdIns(4)P (PI(4)P) or PtdIns(4,5)P_2_ (PIP2). The resulting precipitates and cell lysates (Input) were immunoblotted with indicated antibodies. (**b**) HEK293T cells co-transfected with Myc-CEP164 and GFP-TTBK2 were subjected to immunoprecipitation (IP) with GFP antibody or normal mouse IgG (IgG), in the absence or presence of PI(4)P or PIP2. The resulting precipitates were examined by immunoblotting with indicated antibodies. (**c**) Schematic illustration of the consensus PI-binding motifs in CEP164 and TTBK2. The PI-binding-deficient CEP164 or TTBK2 were generated by mutating or deleting the basic amino acids in these motifs, respectively. (**d**) HEK293T cells co-transfected with indicated combination of wild type (WT) or mutated (9A) Myc-CEP164 and wild type or mutated (ΔPI) GFP-TTBK2 were subjected to IP with anti-GFP antibody. The resulting precipitates and cell lysates (Input) were immunoblotted with indicated antibodies. EV, empty vector; long exp., long exposure; ; short exp., short exposure; α-Tub, α-tubulin.

To fully understand the underlying mechanism, we examined whether TTBK2 or CEP164 can bind PtdIns(4)P and/or PtdIns(4,5)P_2_ *in vitro* by utilizing the PolyPIPosome liposomes^41^. Comparing with the control PolyPIPosomes that do not contain any PI species, PolyPIPosomes containing PtdIns(4)P, but not PtdIns(4,5)P_2_, interacts with both CEP164 and TTBK2 (Fig. 6a). Importantly, excessive PtdIns(4)P but not PtdIns(4,5)P_2_ significantly inhibited the interaction between TTBK2 and CEP164 *in vitro* (Fig. 6b), suggesting that PtdIns(4)P suppresses the TTBK2 recruitment mediated by CEP164. It has been reported that the N terminus of CEP164 binds to the C terminus of TTBK2 (ref. 15). When analysing the peptide sequences of TTBK2 and CEP164, we found consensus PI-binding motifs in CEP164 N terminus and TTBK2 C terminus, respectively (Fig. 6c). These basic motifs are conserved throughout variant vertebrates (Supplementary Fig. 6a,b), suggesting that these motifs may exercise important function. We mutated the 9 lysine in the PI-binding motif of CEP164 to alanine (CEP164-9A) and deleted the basic motif from TTBK2 (TTBK2-ΔPI) (Fig. 6c). Both mutations abolished the interaction with PtdIns(4)P-containing PolyPIPosomes (Supplementary Fig. 6c,d), which confirms that these motifs indeed mediate the binding of PtdIns(4)P to CEP164 and TTBK2, respectively. More importantly, the interactions between the wild-type CEP164 and TTBK2-ΔPI, or between CEP164-9A and the wild-type TTBK2 are both stronger than the interaction between the wild-type proteins; and as expected, CEP164-9A and TTBK2-ΔPI exhibited the strongest interaction (Fig. 6d). These results indicate that both CEP164 and TTBK2 can bind PtdIns(4)P and this binding inhibits the interaction between CEP164 and TTBK2.

**Figure 7:**
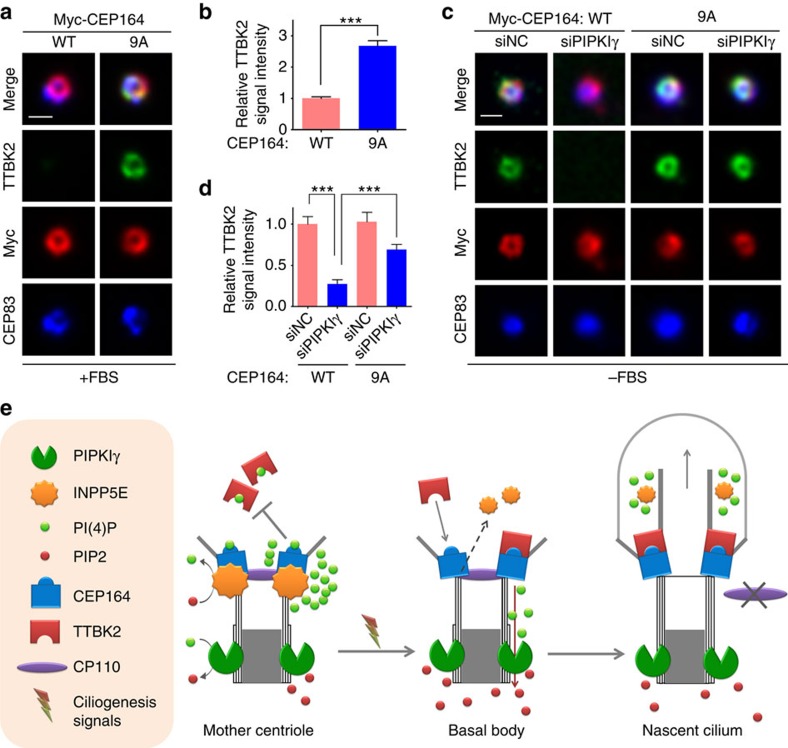
Centrosomal PtdIns(4)P homoeostasis at the M-centriole/basal body. (**a**,**b**) RCTE cells stably expressing GFP-TTBK2 were transfected with wild type (WT) or mutated Myc-tagged CEP164 (9A) for 24 h and subjected to IF microscopy after stained with indicated antibodies. (**b**) The fluorescence intensity of centrosomal TTBK2 in >20 cells of each group quantified from three independent experiments and plotted. (**c**,**d**) RCTE cells stably expressing GFP-TTBK2 were transfected with negative control (siNC) or PIPKIγ siRNA (siPIPKIγ) for 24 h followed by transfection with wild type (WT) or mutant Myc-CEP164 (9A) for another 24 h. After being serum starved for additional 24 h to induce the translocation of TTBK2 to the M-centriole/basal body, these cells were stained with indicated antibodies for IF microscopy. (**d**) The fluorescence intensity of centrosomal TTBK2 was quantified in each group (*n*>20 cells). Results from three independent experiments were analysed and plotted using Excel. Error bars represent s.e.m. ***P<0.001. Scale bars, 0.5 μm. (**e**) Schematic model summarizing the results of current study. In proliferating cells, INPP5E localizes at the distal end of the M-centriole and plays a key role in repelling TTBK2 by maintaining a local PtdIn(4)P (PI(4)P) pool that inhibits the interaction of TTBK2 with CEP164. In quiescent cells when INPP5E leaves the centrosome, the centrosomal PIPKIγ promotes the TTBK2 recruitment and the initiation of ciliary axoneme elongation by consuming the PI(4)P at the centrosome. PIP2, PtdIns(4,5)P_2_.

Consistent with our results from *in vitro* protein–lipid and protein–protein interaction assays, the PtdIns(4)P-binding-deficient CEP164-9A, but not the wild-type CEP164, could recruit the endogenous TTBK2 to the centrosome independent of serum starvation (Fig. 7a,b). In addition, overexpression of CEP164-9A restored the serum-starvation-induced TTBK2 recruitment to the M-centriole/basal body in PIPKIγ-depleted cells (Fig. 7c,d). These results reconcile with our previous observations resulted from manipulating the activity of PIPKIγ or INPP5E, and indicate an interesting possibility that PIPKIγ and INPP5E coordinate PtdIns(4)P homoeostasis at the distal end of the M-centriole/basal body. Insomuch as TTBK2 was established as a critical regulator for the removal of CP110 and axoneme elongation^14^, it is vital to understand how TTBK2 accumulation at the M-centriole/basal body is regulated during the initiating stage of ciliogenesis. Our current results suggest a working model as summarized in Fig. 7e. In non-ciliated cells, INPP5E resides at the distal end of the M-centriole to maintain high level of regional PtdIns(4)P through hydrolyzing PtdIns(4,5)P_2_ presumably generated by PIPKIγ, and these local PtdIns(4)P molecules bind CEP164 and inhibit its interaction with TTBK2. After serum starvation, INPP5E is removed from the basal body through unknown mechanism, which leads to a decrease of local PtdIns(4)P level at the distal end of the basal body. This facilitates the association of TTBK2 with CEP164, and in turn triggers CP110 removal and downstream axoneme elongation.

## Discussion

In spite of only being a small fraction of cellular phospholipids, PIs regulate almost many aspects of cell physiology by spatiotemporally regulating variants cellular processes^19^,^42^. Recently, the role of PI signalling in the context of primary cilia emerges and starts to be appreciated. INPP5E, previously shown to support the stability of pre-existing primary cilia^23^,^33^, is also critical to maintain a PtdIns(4,5)P_2_-low environment in ciliary membrane for the activation of Hedgehog signalling^30^,^31^. Here our results demonstrate a novel role for INPP5E in regulating ciliogenesis. We find that INPP5E resides at the distal end of the M-centriole in non-ciliated cells, in addition to its reported localization in the ciliary lumen in ciliated cells. By keeping a PtdIns(4)P-high microenvironment, active INPP5E at the M-centriole inhibits TTBK2 recruitment and the subsequent CP110 removal and ciliogenesis. These results indicate that the exclusion of INPP5E from the M-centriole is essential for ciliogenesis. Signals initiating ciliogenesis, such as serum starvation, very likely triggers the translocation of INPP5E from the M-centriole to the ciliary lumen. It has been shown that ARL13B, PDE6D and CEP164 are required for the ciliary targeting of INPP5E (ref. 43). Garcia-Gonzalo *et al.*^31^ also showed that TZ components TCTN1 and MKS complex confine INPP5E within the cilium. It would be interesting to investigate whether any of these proteins regulate the dissociation of INPP5E from the M-centriole at the initiating stage of ciliogenesis, and if so, what the underlying mechanism is.

In addition to INPP5E, our results also reveal an innovative role for PIPKIγ in promoting ciliogenesis by depleting centrosomal PtdIns(4)P. This is achieved via a novel mechanism that PtdIns(4)P binds to CEP164 and TTBK2 and inhibits the CEP164–TTBK2 interaction, therefore prevents TTBK2 recruitment. It is worth noting that although artificially decreasing centrosomal PtdIns(4)P by ectopically expressed PIPKIγ is sufficient to promote TTBK2 association with the M-centriole, it fails to completely remove CP110 and initiates axoneme assembly. This suggests that TTBK2, after being recruited to the M-centriole, might need to be further activated by certain PIPKIγ-independent mechanism to achieve the complete removal of CP110 that is required for axoneme elongation. Nevertheless, our results indicate that PIPKIγ and INPP5E coordinates the centrosomal homoeostasis of PtdIns(4)P during ciliogenesis. Given the importance of PI signalling in ciliogenesis initiation identified here and the identification of various PI 5-phosphatases (such as INPP5E, INPP5B and OCRL) as causal loci for ciliopathies, we reason that PIPKIγ malfunction may also be correlated with ciliopathies. A kinase-dead mutation in PIPKIγ leads to neurodegeneration in human^44^ and PIPKIγ-null mice that exhibit embryonic lethality at E11.5 with defects in neuronal tube closure^45^, both of which are likely ciliopathy phenotypes. Due to the existence of multiple PIPKIγ splicing isoforms, further *in vivo* investigations are needed to dissect the exact correlation between each PIPKIγ isoform with primary cilia and ciliopathies.

Here we report a functional centrosomal PtdIns(4)P pool that is coordinated by INPP5E and PIPKIγ. All of the PtdIns(4)P-enriched endomembranes, such as the Golgi complex, Golgi-derived carriers, as well as the recycling endosome^46^,^47^,^48^, likely contribute to ciliogenesis by supporting IFT20-dependent vesicle transport^49^ or composing the ciliary vesicles that attach to the distal end of the M-centriole/basal body to form the ciliary membrane^50^. Since we were not able to co-stain any membrane marker with the PtdIns(4)P antibody using immunofluorescence microscopy, whether the centrosomal PtdIns(4)P we observed is PtdIns(4)P-containing vesicles should be confirmed by using immunoelectron microscopy. It is also possible that unknown ciliary PI-transfer protein(s)^51^ shuttles PtdIns(4)P and/or PtdIns(4,5)P_2_ between the distal and proximal ends of the M-centriole where INPP5E and PIPKIγ reside, respectively.

Similar as we reported here and previously^32^, Chavez *et al.*^30^ also observed PIPKIγ at the ciliary base. However, we failed to detect outstanding PtdIns(4,5)P_2_ accumulation at the centrosome or basal body using both specific antibody and biosensor, although our results indicate that PIPKIγ should generate PtdIns(4,5)P_2_ by phosphorylating the centrosomal PtdIns(4)P during ciliogenesis initiation. This could be due to inappropriate technique, or the PtdIns(4,5)P_2_ produced by PIPKIγ is quickly consumed or masked by centrosomal/ciliary base PtdIns(4,5)P_2_ effector or binding proteins like MARCKS^52^. In addition to supporting ciliogenesis, PIPKIγ in ciliated cells may help maintain the proximal PtdIns(4,5)P_2_-containing domain of the ciliary membrane, which is indispensible for ciliary signalling by regulating the transport of ciliary cargos, such as in the case of TULP3-mediated transport of Gpr161 and hedgehog signalling inhibition^31^. Moreover, multiple TZ proteins contain C2 domain^13^ that could bind PIs^53^. Whether these proteins are regulated by PI signalling should be investigated. Interestingly, a recent work indicates that C2 domain-containing TZ protein MKS5 establishes a TZ zone that limits the abundance of PtdIns(4,5)P_2_ within cilia and is required for ciliary signalling^54^, indicating complex interactions between PIs, PI enzymes and PI effectors in the context of primary cilium. After all, how cilium-associated PI metabolizing enzymes orchestrate the dynamic, spatiotemporally regulated homoeostasis of variant PIs to support ciliogenesis and cilium function is a fascinating question to explore in the future. Our current work demonstrates the PIPKIγ-INPP5E signalling nexus and the novel role of PtdIns(4)P homoeostasis in the initiation of ciliogenesis, which provides attractive targets for future small-molecule intervention of human ciliopathies related to PIPKIγ and/or INPP5E.

## Methods

### Antibodies

Rabbit polyclonal antibodies against the following proteins were used: CEP83 (HPA038161), CEP164 (SAB3500022) and TTBK2 (HPA018113) (Sigma); CP110 (A301-343A, Bethyl Laboratories); CEP135 (ab75005), Actin (ab8227) (Abcam); FBF1 (11531-1-AP), TCTN1 (15004-1-AP), SCLT1 (14875-1-AP), MKS1 (16206-1-AP), INPP5E (17797-1-AP) and IFT88 (13967-1-AP) (Proteintech). Rabbit antibodies against PIPKIγ, PIPKIα and PIPKIβ were described previously^32^. Mouse antibodies against the following proteins were used: Centrin (20H5) (kindly provided by Dr Jeffrey Salisbury, Mayo Clinic); polyglutamylated tubulin (ALX-804-885-C100, Enzo Life Sciences); ODF2 (H00004957-M01, Abnova); acetylated α-tubulin (T7451), α-tubulin (T9026), γ-tubulin (T6557), FLAG (F1804) and HA (H3663) (Sigma); GFP (A-11120, Invitrogen); PI(4)P (Z-P004) and PI(4,5)P2 (Z-P045) (Echelon). Mouse monoclonal NPHP1 antibody (IgG 1) was generated at Abmart. For western blots, we used a 1:5,000 dilution for antibodies except for those against α-tubulin and actin (1:20,000). For immunofluorescence microscopy, a 1:500 dilution for antibodies was used except for those against NPHP1 (1:50), acetylated α-tubulin, polyglutamylated tubulin, centrin (1:2,000), PtdIns(4)P and PtdIns(4,5)P_2_ (1:100). Uncropped western blots are shown in Supplementary Fig. 7.

### DNA constructs and siRNAs

The GFP-TTBK2 construct was kindly provided by Dr Kathryn Anderson (Sloan-Kettering Institute, New York, NY). The Flag-INPP5E construct was a kind gift from Dr Seongjin Seo (University of Iowa, Iowa City, IA). We obtained the following constructs from Addgene: Myc-CEP164 (Addgene plasmid #41148, from Dr Erich Nigg), GFP-ING2 (pHD) (Addgene plasmid #21589, from Dr Junying Yuan), GFP-FYVE (EEA1) (Addgene plasmid #36096, from Dr Scott Emr). We sub-cloned the FYVE(EEA1) into pEGFP-C1 vector. PIPKIγ and PIPKIγΔCT were amplified from cDNAs and sub-cloned into pCMV-HA vector^32^. Inactive PIPKIγ (D253/316A)^55^,^56^, INPP5E (D512/515W)^33^ and PI-binding-deficient CEP164 and TTBK2 mutants (Myc-CEP164-9A and GFP-TTBK2-ΔPI) were generated by using a QuikChange Site-Directed Mutagenesis Kit (Agilent Technologies). All constructs were verified by DNA sequencing.

siRNA duplexes were introduced into cells with Lipofectamine RNAiMAX (Invitrogen), following the manufacturer's manual. Typically after 72 h of siRNA transfection, cells were collected for further analysis by western blot or immunofluorescence microscopy. siRNA targeting INPP5E (ON-TARGETplus SMARTpool, #L-020852-00-0005) and RNAi Negative Control were purchased from GE Healthcare Dharmacon. PIPKIγ, PIPKIα and PIPKIβ siRNA oligonucleotides were obtained from Invitrogen. PIPKIγ-O1 and PIPKIγ-O2 are siRNAs targeting human PIPKIγ.

PIPKIγ-O1, 5′-GCGTGGTCAAGATGCACCTCAAGTT-3′;

PIPKIγ-O2: 5′-GCTACTACATGAACCTCAACCAGAA-3′.

mouse PIPKIγ: PIPKIγ-O3, 5′-GCGAGAGAGAGGATGTGCAGTATGA-3′.

Human PIPKIα: 5′-TTGAAAGGTGCCATCCAGTTAGGCA-3′;

Human PIPKIβ: 5′-CAGCAAAGGGTTACCTTCCAGTTCA-3′;

### Cell culture and transfection

NIH3T3 and HEK293T cells were cultured in DMEM containing 10% foetal bovine serum (FBS). hTERT-RPE-1 (RPE-1), inner medullary collecting duct (IMCD3) and RCTE cells were cultured in DMEM/F-12 containing 10% FBS.

For plasmid transfection, X-tremeGENE 9 (Roche) were used following the manufacturer's manual. To generate RCTE cell line stably expressing GFP-TTBK2, RCTE cells were passed into complete growth medium containing 800 μg ml^−1^ G418 (MediaTech) for selection, and maintained in medium containing 400 μg ml^−1^ G418.

### Immunofluorescence microscopy

For indirect immunofluorescence, cells were grown on glass coverslips and fixed with ice-cold methanol or paraformaldehyde for 10 min, then permeabilized and immune-stained with appropriate antibodies. Fluorescence images were acquired using Nikon TE2000-U with Metamorph software (Molecular Devices). Immunofluorescence staining of PtdIns(4)P and PtdIns(4,5)P_2_ was performed following published methods^30^,^31^,^57^. The specificity of the PtdIns(4)P and PtdIns(4,5)P_2_ antibodies was verified by pre-absorbing the antibody with PolyPIPosomes containing 5% of PtdIns(4)P or PtdIns(4,5)P_2_ (Echelon) for 1 h at room temperature before incubation with samples.

Three dimensional structured illumination microscopy was performed following standard protocol. Briefly, an ELYRA Super-resolution Microscopy system (Zeiss) equipped with an alpha ‘Plan-Apochromat' 100 × /1.46 Oil DIC oil immersion objective and an Andor iXon 885 EMCCD camera was used to acquire raw images following standard protocols. Sections were acquired at 0.125-mm z-steps. Colour channels were aligned using alignment parameter from control measurements with 0.5-μm diameter multispectral fluorescent beads (Zeiss). Structured illumination reconstruction and image processing were performed with the ZEN software package (Zeiss). Final image processing was done using Adobe Photoshop (Adobe).

### Immunoprecipitation and PolyPIPosome pull-down assay

Immunoprecipitation was performed using HEK293T cell lysate 48 h post transfection, in IP buffer (20 mM Hepes-KOH, pH 7.2, 10 mM KCl, 1.5 mM MgCl_2_, 1 mM EDTA, 1 mM EGTA, 150 mM NaCl, 0.5% NP-40), with Complete Protease Inhibitor Cocktail (Roche) and PhosSTOP Phosphatase Inhibitor Cocktail (Roche) added following the manufacturer's manuals.

For immunoprecipitation assay in the presence of PIs, HEK293T cells co-transfected with Myc-CEP164 and GFP-TTBK2 were subjected to immunoprecipitation with GFP antibody and Protein G Sepharose 4 Fast Flow beads (GE Healthcare) overnight at 4 °C. After being washed, the protein-bound beads were incubated with 25 μM diC8-PtdIns(4)P or diC8-PtdIns(4,5)P_2_ (Echelon) for additional 3 h at 4 °C. The resulting precipitates were examined by immunoblotting with appropriate antibodies.

For PolyPIPosome pull-down assay, HEK293T cells were transfected with Myc-CEP164 or GFP-TTBK2 for 48 h, and lysed in PolyPIPosome-binding buffer (50 mM Tris, pH 7.4, 150 mM NaCl, 1 mM EGTA, 1 mM MgCl_2_, 1 mg ml^−1^ bovine serum albumin, 0.2 mM CaCl_2_, 5 mM KCl, 0.05% NP-40) on ice. The cell lysate was incubated with 50 μM biotin-tagged control or PI-containing PolyPIPosomes (containing 5% variant PI, Echelon Biosciences) overnight at 4 °C, then incubated with Streptavidin Agarose beads (Thermo Fisher Scientific) for additional 1 h. After being washed for three times with PolyPIPosome-binding buffer, the samples were analysed by immunoblotting with appropriate antibodies.

### Electron microscopy

For transmission electron microscopy, IMCD3 cells treated with PIPKIγ siRNA, or negative control siRNA were grown on coverslips. After 72 h, the cells were serum starved to induce primary ciliogenesis for additional 24 h. The cells were then fixed overnight using Trump's fixative (Electron Microscopy Sciences), scraped off the coverslips and spun into pellets. Embedding and thin sectioning of cell samples were performed according to standard procedures at the Electron Microscopy Core Facility, Mayo Clinic. Specimens were observed under a JEOL 1400 transmission electron microscope (JEOL) operating at 80 kV.

For scanning electron microscopy, IMCD3 cells treated with appropriate siRNAs were grown on coverslips. After 72 h, cells were serum starved for additional 24 h to induce primary ciliogenesis. These cells were then fixed overnight in Trump's fixative and processed at the Mayo Clinic Electron Microscopy Core Facility according to standard procedures. Specimens were observed under a scanning electron microscope (Hitachi, S-4700).

### Dye-filling assay

A dye-filling assay was performed using an established protocol^58^. Briefly, worms were washed off the culturing plate with M9 buffer (3 g l^−1^ KH_2_PO_4_, 6 g l^−1^, Na_2_HPO_4_, 5 g l^−1^ NaCl and 1 mM MgSO_4_), collected by centrifugation at 500 g for 1 min, washed once with M9 buffer, and then incubated in diluted DiI dye (Molecular Probes, D-282; 1:200 dilution in M9 buffer of the 2 mg ml^−1^ stock solution in dimethyl formamide) for 1 h at room temperature. After incubation, worms were washed at least three times with M9, transferred to an NGM plate without a bacterial lawn, and observed under a fluorescence microscope (Zeiss, M2Bio).

### Statistical analyses

Results are presented as mean plus s.d. or s.e.m., as specified in each figure legend. Significance was calculated by double-tailed *t*-test using Excel (Microsoft). *P<0.05, **P<0.01 and ***P<0.001 were considered as statistically significant differences.

## Additional information

**How to cite this article**: Xu, Q. *et al.* Phosphatidylinositol phosphate kinase PIPKIγ and phosphatase INPP5E coordinate initiation of ciliogenesis. *Nat. Commun.* 7:10777 doi: 10.1038/ncomms10777 (2016).

## Supplementary Material

Supplementary Information Supplementary Figures 1-7
