# Supplementary material for: Phosphatidylinositol phosphate kinase PIPKIγ and phosphatase INPP5E coordinate initiation of ciliogenesis
Source: Nat Commun. 2016 Feb 26;7:10777. doi: 10.1038/ncomms10777 (PMC4773430; doi:10.1038/ncomms10777)
Supplement: Supplementary Information — Supplementary Figures 1-7 [file ncomms10777-s1.pdf]

Xu et al., Supplementary Figures 1-7

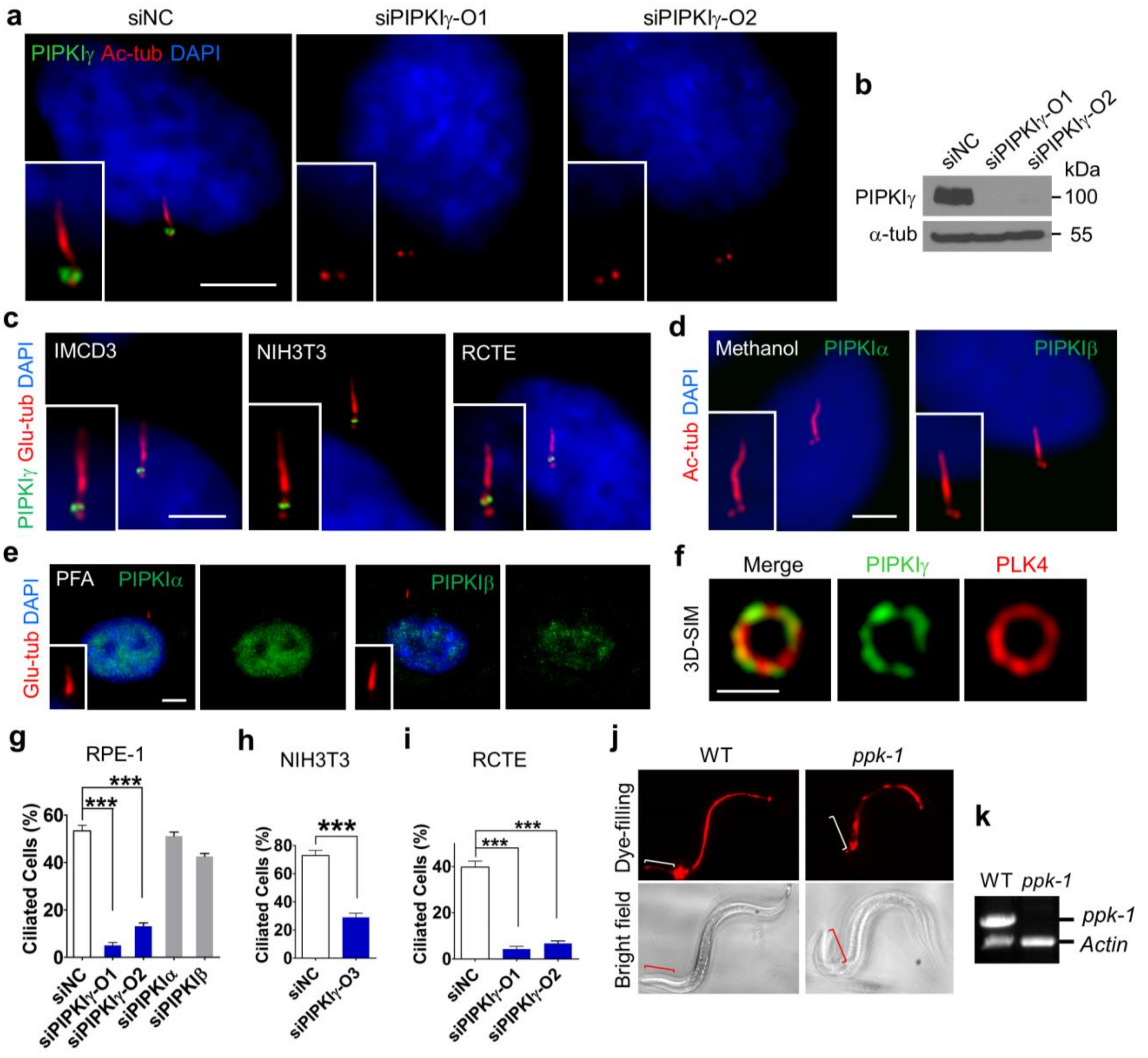

Supplementary Figure 1. PIPKIγ is required for ciliogenesis. (**a**) PIPKIγ localizes at the basal body of primary cilium. RPE-1 cells treated with two siRNAs targeting to human PIPKIγ (siPIPKIγ-O1, siPIPKIγ-O2) or negative control siRNA (siNC) were serum starved to induce primary ciliogenesis and stained with appropriate antibodies. (**b**) Knockdown of PIPKIγ was validated by immunoblotting. (**c**) PIPKIγ exhibits basal body localization in multiple types of ciliated cells. (**d, e**) PIPKIα and PIPKIβ antibodies yielded no cilium-related signal. Serum starved RPE-1 cells were fixed with methanol (**d**) or

paraformaldehyde (PFA) (**e**) and stained with appropriate antibodies. (**f**) PIPKIγ forms a ring-like structure at the ciliary base. RPE-1 cells co-stained with anti-PIPKIγ and anti-PLK4 antibodies were examined by 3D-SIM microscopy. (**g**-**i**) PIPKIγ depletion substantially inhibits primary ciliogenesis in RPE-1, NIH3T3, and RCTE cells. (**g**) RPE-1 cells treated with negative control (siNC) or siRNAs against PIPKIγ (siPIPKIγ-O1 and siPIPKIγ-O2), PIPKIα (siPIPKIα), or PIPKIβ (siPIPKIβ) were serum starved to induce ciliogenesis and stained with anti-acetylated α-tubulin antibody (Ac-tub). (**h**) NIH3T3 cells were treated with siNC or mouse PIPKIγ-specific (siPIPKIγ-O3) siRNAs. (**i**) RCTE cells treated with siNC or two PIPKIγ-specific siRNAs were analyzed as described in (**a**). The percentage of ciliated cells (n > 200) in each group was quantified from at least three independent experiments and plotted. Error bars represent s.d. ***P < 0.001. (**j**) Severe ciliary defect was observed in *ppk-1*-null *C. elegans*. With dye-filling assay, *ppk-1*-null worms exhibited complete dye-filling minus phenotype. Brackets indicate ciliated Amphid neurons. (**k**) Genotyping results of *ppk-1*-null worms. *Actin* was used as a loading control. Nuclei were visualized by DAPI staining (blue). Insets show magnified images of primary cilia. Ac-tub, acetylated α-tubulin. Glu-tub, polyglutamylated tubulin. α-tub, α-tubulin. Scale bars are 0.5 μm in (**f**) and 5 μm in other panels.

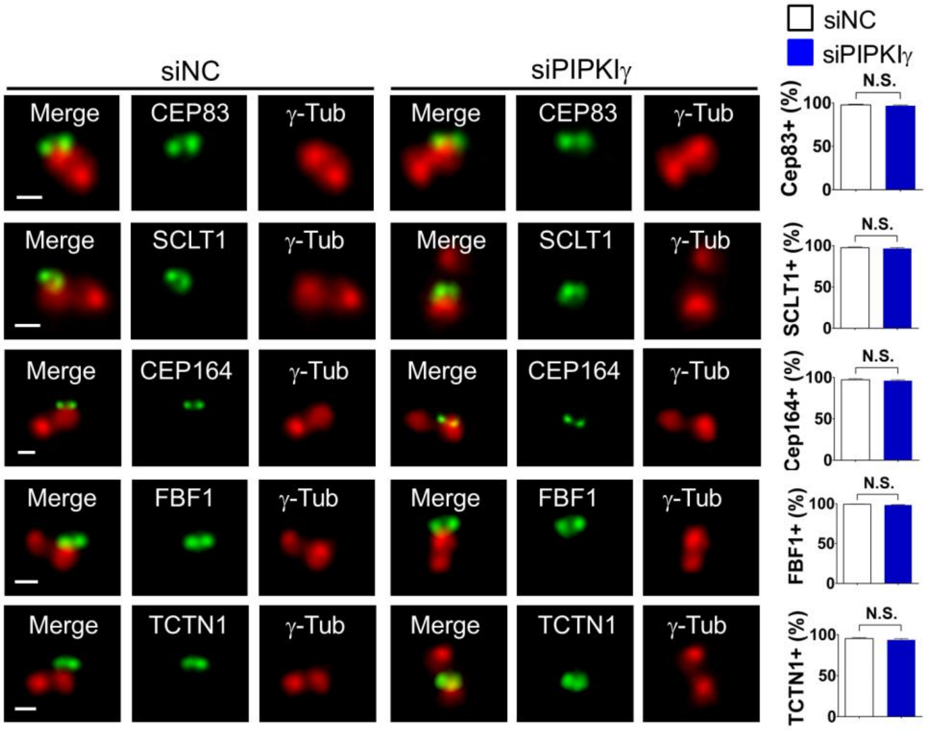

Supplementary Figure 2. PIPKIγ functions downstream of basal-body membrane docking and upstream of TZ formation. RPE-1 cells treated with negative control (siNC) or PIPKIγ (siPIPKIγ) siRNA were serum starved and stained with the appropriate antibodies. Percentage of cells ($n > 200$) showing positive staining of indicated proteins was quantified from at least three experiments. Error bars represent standard deviation (s.d.). N.S., no statistically significant difference. γ-tub, γ-tubulin. Scale bars, 0.5 μm.

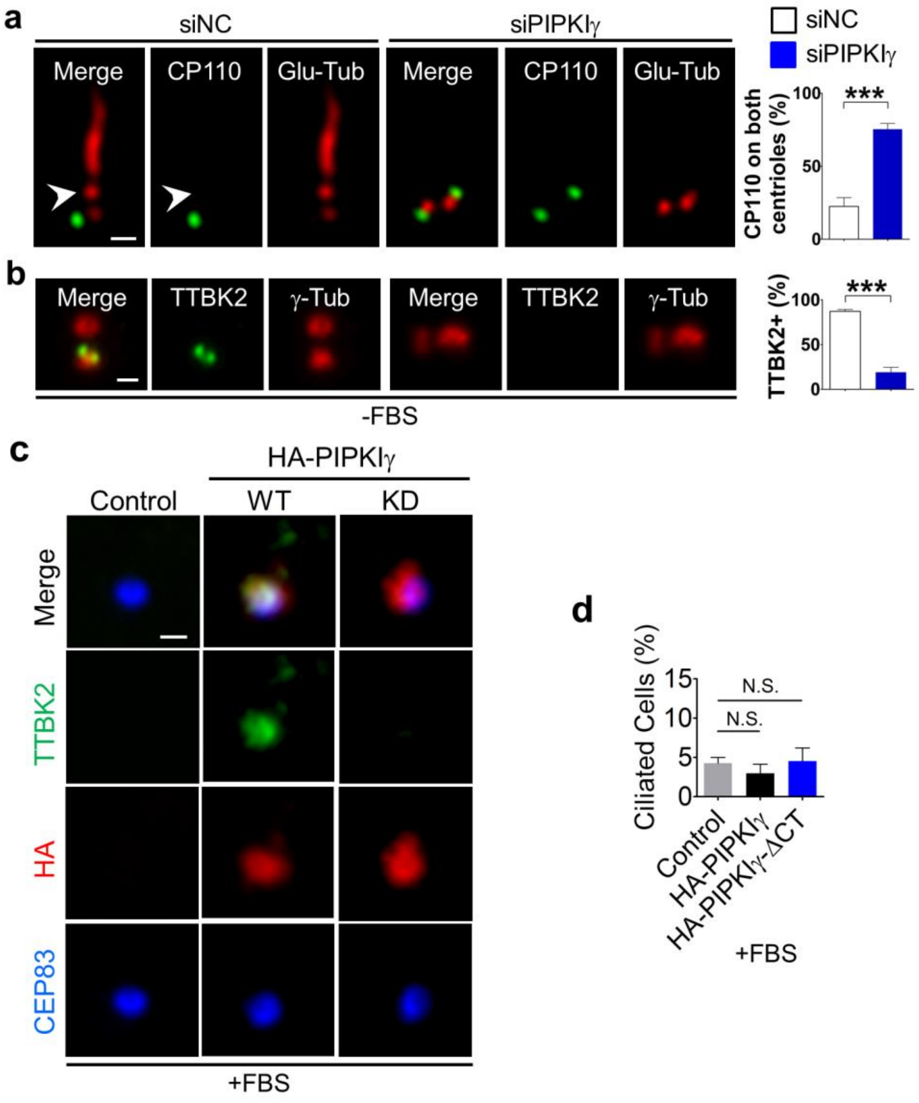

Supplementary Figure 3. PIPKIγ promotes the recruitment of TTBK2 to the M-centriole/basal body. (**a**) CP110 retained at the M-centriole in serum-starved, PIPKIγ-depleted RPE-1 cells. Arrowheads point to the basal body. (**b**) TTBK2 recruitment to the M-centriole induced by serum starvation was impaired in RPE-1 cells when PIPKIγ was knocked down. Percentage of cells (n > 200) exhibiting CP110 on both centrioles (**a**), or TTBK2 at the M-centriol/basal body (**b**) was quantified from at least three independent experiments. Glu-tub, polyglutamylated tubulin. γ-tub, γ-tubulin. (**c**) PIPKIγ overexpression promotes TTBK2 recruitment to the centrosome in a PIPKIγ kinase activity dependent manner. RCTE cells stably expressing GFP-TTBK2 were transfected with empty vector

(Control), wild-type (WT), or kinase-dead (KD) HA-PIPKIγi3 for 8 h without serum starvation. Cep83 was used as a centrosome marker. (**d**) RCTE cells were transfected with empty vector (Control), HA-PIPKIγ, and HA-PIPKIγΔCT. The percentage of ciliated cells (n > 20) in each group was quantified from at least three independent experiments and plotted. Error bars represent standard deviation (s.d.). ***P < 0.001. N.S., no statistically significant difference. Scale bars, 0.5 μm.

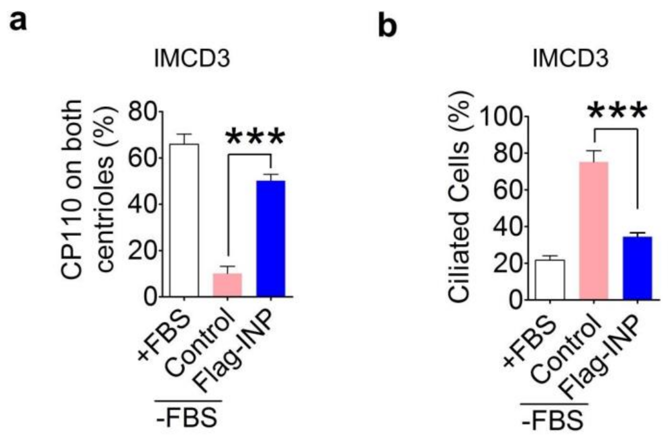

Supplementary Figure 4. INPP5E inhibits ciliogenesis by suppressing CP110 removal from the basal body. IMCD3 Cells transfected with empty vector (Control), or Flag-INPP5E were serum starved and stained with indicated antibodies. Percentage of cells with CP110 on both centrioles (**a**, n > 100) and ciliated cells (**b**, n > 200) was quantified from at least three independent experiments. Error bars represent s.d. ***P<0.001.

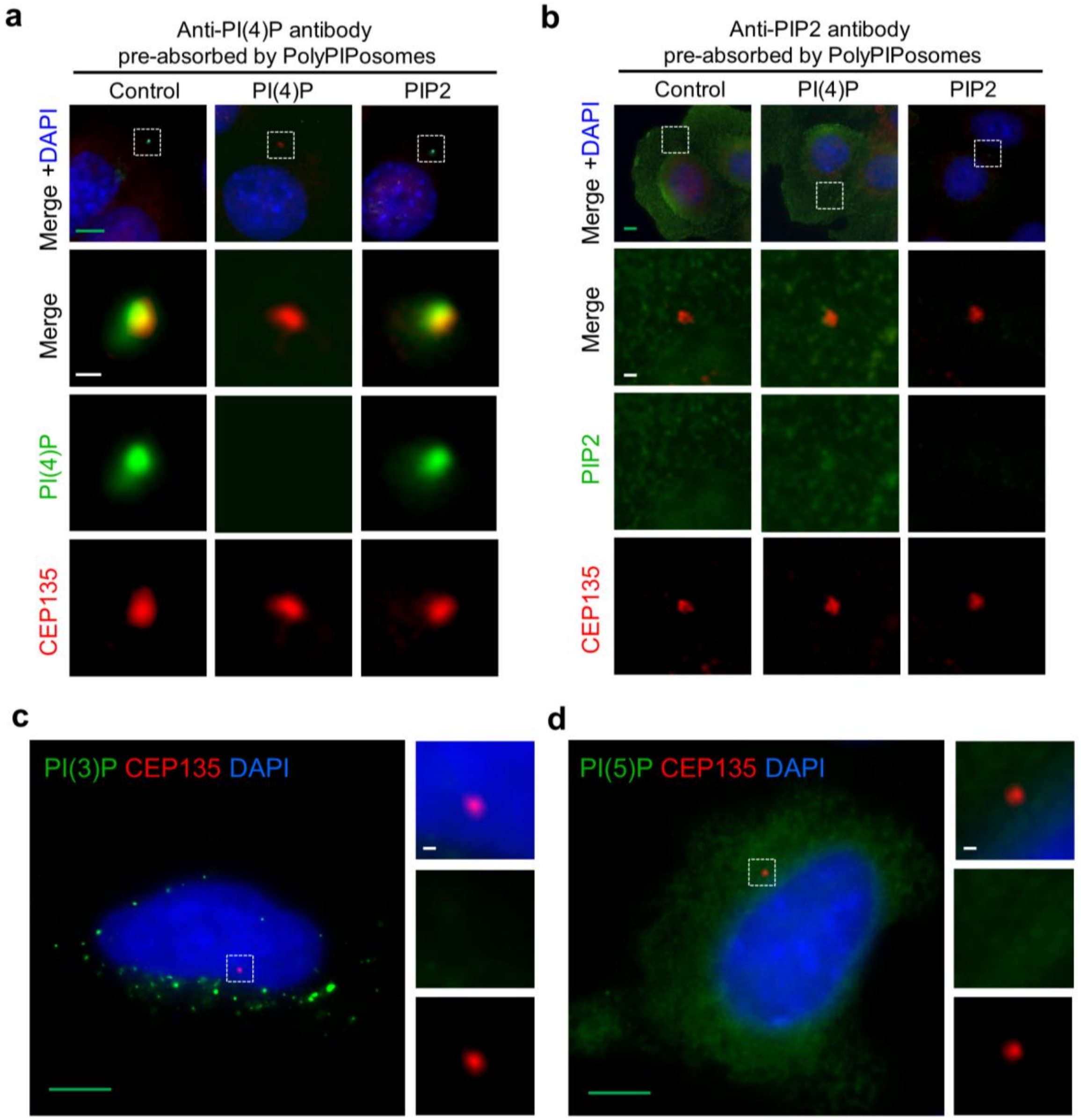

Supplementary Figure 5. Localization of PI variants in cell. (**a, b**) IMCD3 cells were stained with indicated antibodies, and nuclei were visualized by DAPI staining. Anti-PtdIns(4)P (PI(4)P) (**a**) and anti-PtdIns(4,5)P_2 (PIP2) (**b**) antibodies were pre-absorbed with control PolyPIPosomes (Control), or PolyPIPosomes containing 5% of PI(4)P or PIP2 before adding onto samples for immunostaining. (**c, d**) IMCD3 cells transfected with the PI(3)P probe GFP-FYVE, or the PI(5)P probe GFP-PHD were processed for immunofluorescence microscopy. Scale bars: green, 5 μm; white, 0.5 μm.

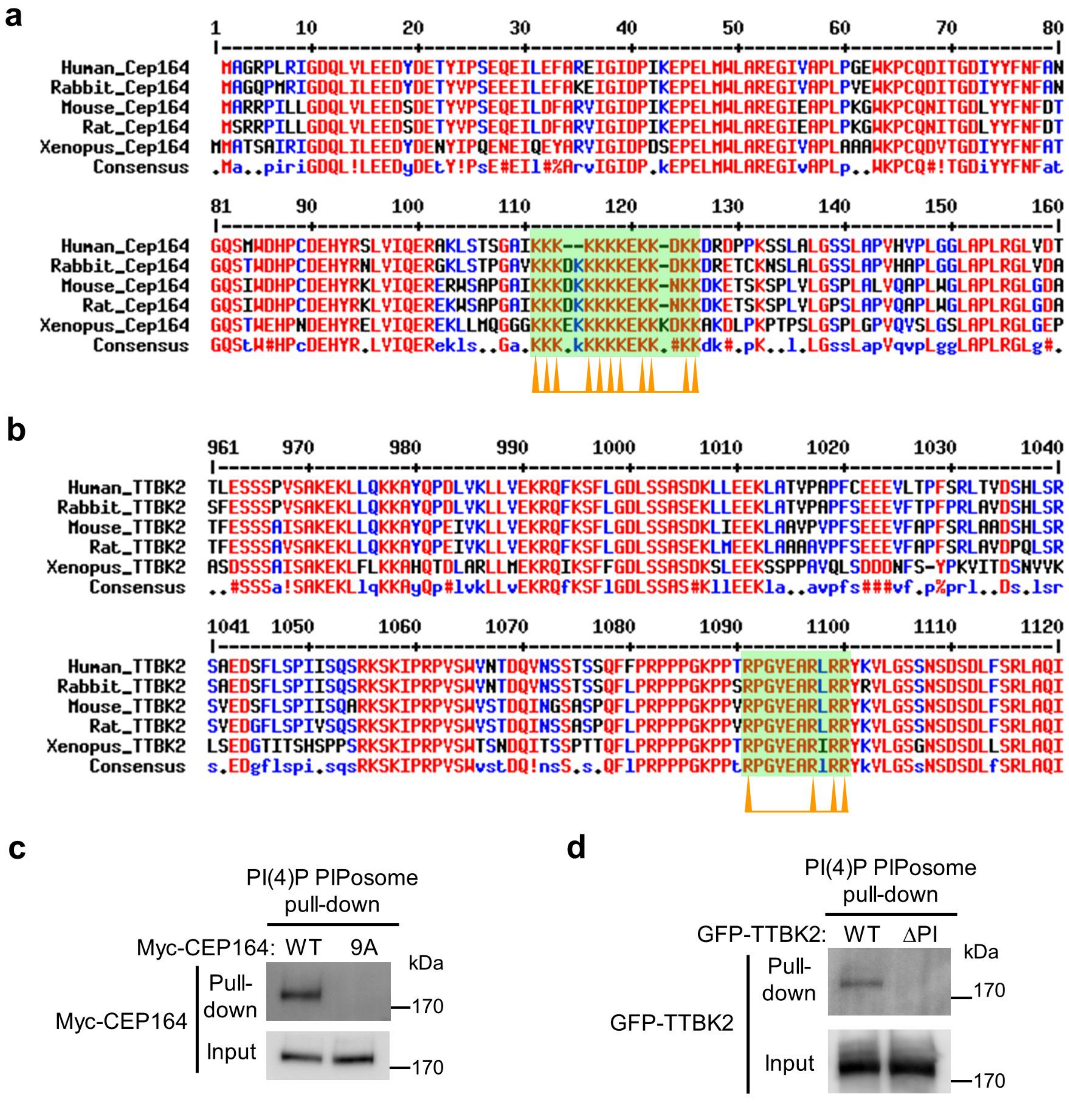

Supplementary Figure 6. CEP164 and TTBK2 contain evolutionarily conserved PI binding motifs. The protein sequences of Cep164 (**a**) and TTBK2 (**b**) orthologs were analyzed by MULTALIN alignment software. Evolutionarily conserved PI-binding motifs with lysine/arginine-rich patches were highlighted. (**c, d**) PolyPIPosome pull-down assay. HEK293T Cells transfected with

wild-type (WT) Myc-CEP164 or GFP-TTBK2, or mutated Myc-CEP164 (9A) and GFP-TTBK2 (ΔPI) were subjected to pull-down assay with PolyPIPosomes containing PI(4)P. The resulting precipitates and cell lysates (Input) were immunoblotted with indicated antibodies.

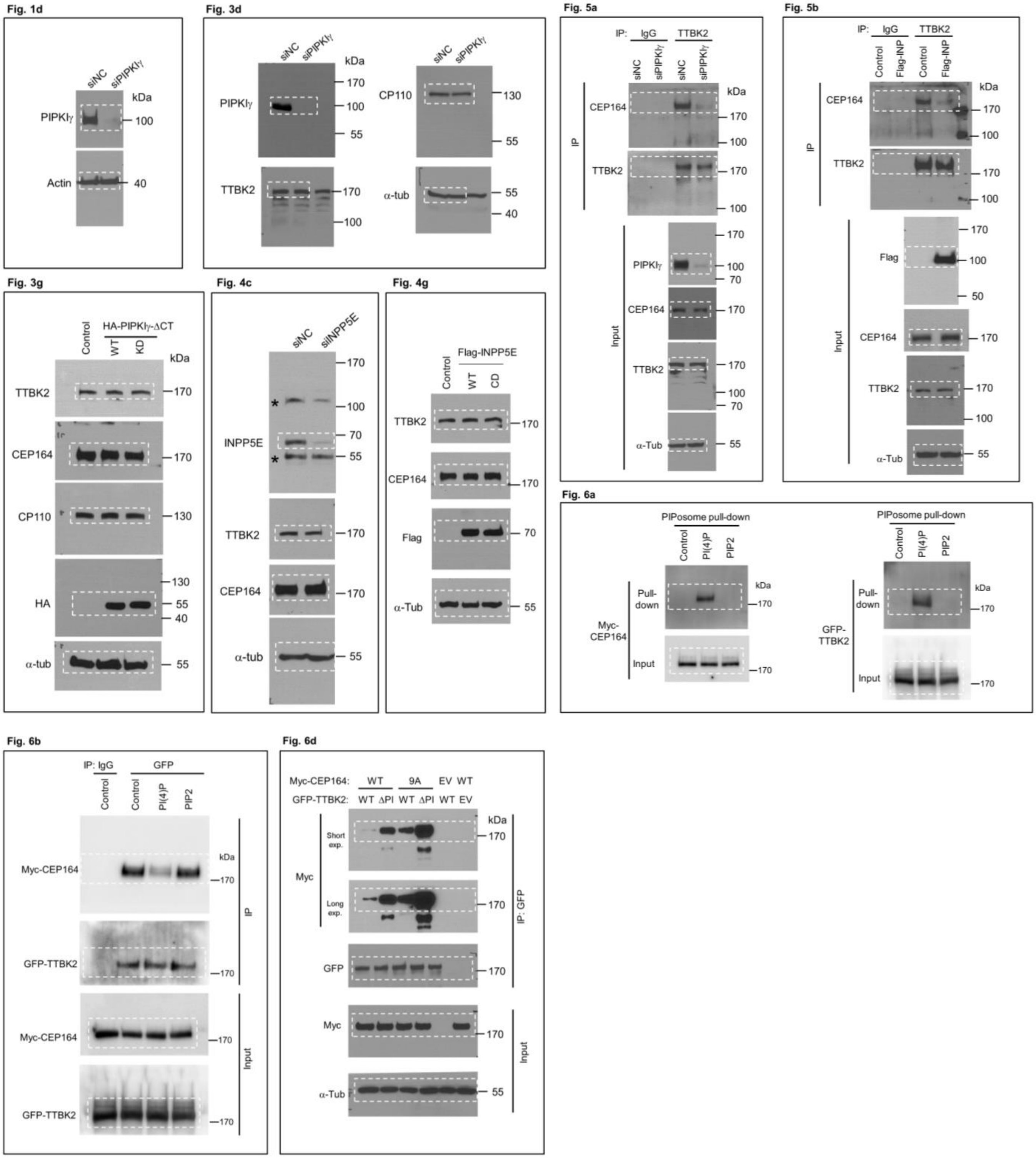

Supplementary Figure 7. Uncropped images of immunoblotting results for Figs. 1-7.
